# The strategy for enhancing temozolomide against malignant glioma

**DOI:** 10.3389/fonc.2012.00098

**Published:** 2012-08-14

**Authors:** Mitsutoshi Nakada, Takuya Furuta, Yutaka Hayashi, Toshinari Minamoto, Jun-ichiro Hamada

**Affiliations:** ^1^Department of Neurosurgery, Kanazawa UniversityKanazawa, Ishikawa, Japan; ^2^Division of Translational and Clinical Oncology, Cancer Research Institute, Kanazawa UniversityKanazawa, Ishikawa, Japan

**Keywords:** temozolomide, glioma, MGMT, chemosensitivity, interferon-β, levetiracetam, resveratrol, valproic acid

## Abstract

A combined therapy of the alkylating agent temozolomide (TMZ) and radiotherapy is standard treatment, and it improves the survival of patients with newly diagnosed glioblastoma (GBM). The DNA repair enzyme O^6^-methylguanine-DNA methyltransferase (MGMT) removes the most cytotoxic lesions generated by TMZ, O^6^-methylguanine, establishing MGMT as one of the most important DNA repair mechanisms of TMZ-induced DNA damage. Thus, the expression of MGMT, its activity, and its promoter methylation status are associated with the response of GBM to TMZ, confirming that MGMT promotes clinical resistance to TMZ. Previous studies have shown that a variety of drugs such as interferon-β (IFN-β), levetiracetam (LEV), resveratrol, and valproic acid (VAP) increased the sensitivity of TMZ through MGMT-dependent or MGMT-independent mechanisms. In this review, we describe drugs and promising molecules that influence the responsiveness of GBM to TMZ and discuss their putative mechanism of action. In MGMT-positive GBMs, drugs that modulate MGMT activity could enhance the therapeutic activity of TMZ. Thus, administration of these drugs as an adjunct to TMZ chemotherapy may have clinical applications in patients with malignant gliomas to improve the outcome.

## Introduction

Glioblastoma (GBM) is the most common and lethal among all gliomas. The current standard of care includes surgery followed by concomitant radiation and chemotherapy with the oral DNA-alkylating agent with good penetration into the blood-brain barrier, temozolomide (TMZ). O^6^-methylguanine-DNA methyltransferase (MGMT) repairs the most cytotoxic lesions generated by TMZ, O^6^-methylguanine. Accordingly, increased expression of MGMT is one of the most robust predictors of the TMZ response in malignant glioma cells. The most important mechanism of silencing of the MGMT gene is methylation of its promoter, resulting in the loss of MGMT expression and diminished DNA repair activity. Therefore, the epigenetic silencing of MGMT gene by its promoter methylation has been shown to be a useful predictor of responsiveness of GBM patients to TMZ (Hegi et al., 2005).

Because tumor cells that express MGMT are more resistant to TMZ, targeting the MGMT activity can enhance the therapeutic efficiency of TMZ. Recently, a phase III clinical trial examined the effects of dose-dense TMZ as a strategy to deplete MGMT and enhance the outcome in GBM patients (RTOG0525, ASCO 2011). In addition, preclinical studies and clinical trials are investigating whether it is possible to increase the anticancer potency of TMZ by combining it with other pharmacological agents (Goellner et al., 2011; Agnihotri et al., 2012). In this review, we discuss drugs that modulate the therapeutic efficacy of TMZ via MGMT-dependent or MGMT-independent mechanisms.

## MGMT-dependent mechanism

Methylation of the MGMT promoter in GBM patients correlates with increased susceptibility of the tumor to the alkylating agent therapy. Several drugs have been reported to induce methylation of the MGMT promoter, resulting in increased TMZ cytotoxicity.

### O6-benzylguanine (O6-BG)

O^6^-benzylguanine (O^6^-BG), a low-molecular-weight substrate, can irreversibly inactivate MGMT, by competing with O^6^-methylguanine. *in vitro* (Dolan et al., 1991; Bobola et al., 1996) and *in vivo* (Friedman et al., 1995) studies confirmed the O^6^-BG increases the therapeutic activity of TMZ. Accordingly, a phase II clinical trial revealed limited benefits with high incidence of bone marrow suppression when patients with TMZ-resistant anaplastic gliomas were treated with both O^6^-BG and TMZ. However, it was disappointing that no significant restoration of TMZ sensitivity occurred in patients with TMZ-resistant GBM (Quinn et al., 2009). Because of the limited response seen in GBM patients, alternative dosing regimens should be investigated in order to optimize combination treatment with TMZ and O^6^-BG.

### Interferon-β

Interferon-β (IFN-β), which belongs to type I IFNs, was first discovered on the basis of its antiviral activities. Subsequently, it was shown to exhibit pleiotropic biological activities including immunomodulatory activity; antiangiogenic activity; and direct antitumor effects, e.g., growth inhibition and apoptosis (Borden et al., 2007). IFN-β markedly enhanced sensitivity to TMZ via downregulation of MGMT transcription (Natsume et al., 2005; Yoshino et al., 2009). The results of the study suggest that compared to TMZ-based chemotherapy plus radiotherapy, chemotherapy with IFN-β and TMZ and concomitant radiotherapy further improve the clinical outcomes of patients with malignant gliomas. A multicenter phase I clinical trial established that therapy with IFN-β and TMZ is safe, well tolerated, and prolongs survival of patients with GBM (Wakabayashi et al., 2011). The median survival time (MST) of patients who underwent the IFN-β and TMZ combination therapy was significantly longer (19.9 months) than that of patients treated with TMZ alone (12.7 months). Remarkably, the MST of patients whose tumors had the unmethylated MGMT promoter was prolonged to 17.2 months when receiving TMZ with IFN-β, compared to 12.5 months in patients receiving TMZ alone. Taken together, IFN-β increased the therapeutic efficiency of TMZ in cases of newly diagnosed primary GBM, particularly in patients with the unmethylated MGMT promoter (Motomura et al., 2011). A prospective randomized control trial to compare the clinical outcomes of newly diagnosed GBM patients treated with TMZ alone or with TMZ and IFN-β combination therapy is ongoing.

### Levetiracetam

**Figure 1:**
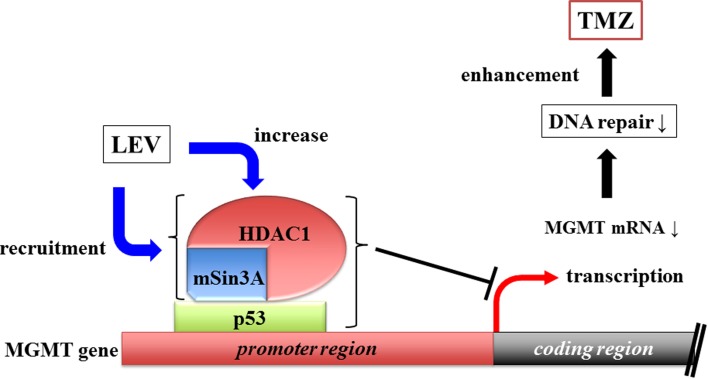
**Levetiracetam increases HDAC1 transcription and recruit HDAC1/mSin3A corepressor, which binds MGMT promoter region intermediated by p53.** This complex of three components inhibits MGMT transcription.

Some antiepileptic drugs (AEDs) have the ability to inhibit histone deacetylase (HDAC) activity. HDAC inhibitors (HDACi) have transcriptional regulatory activity (Strahl and Allis, 2000), suggesting that they can influence TMZ efficacy by modulating the expression of MGMT. HDACs are attractive targets in cancer therapy because their inhibition can induce cell differentiation, growth arrest, and apoptosis (Li et al., 2005). Levetiracetam (LEV), a relatively new AED, does not directly inhibit HDAC activity *in vitro* at therapeutic concentration (Eyal et al., 2004). Instead, LEV increases the transcription of HDAC1 and recruits the HDAC1/mSin3A corepressor complex to the p53-binding site in the MGMT promoter (Bobustuc et al., 2010), thus silencing MGMT (Figure 1).

In contrast, LEV increases MGMT transcription in normal astrocytes (Bobustuc et al., 2010) and exerts a neuroprotective role through free-radical scavenging activity (Ueda et al., 2009). Moreover, LEV reduces the extent of inflammation and neuronal death by inducing the expression of neurotrophic factors and inducible nitric oxide synthase (iNOS) (Cardile et al., 2003). All this activities may contribute to the ability of LEV to prevent radiochemotherapy-induced nerve damage.

Taken together, TMZ-induced cytotoxicity in GBM patients who express high levels of MGMT may be enhanced by concomitant administration of LEV with little adverse effects.

## MGMT-independent mechanism

**Figure 2:**
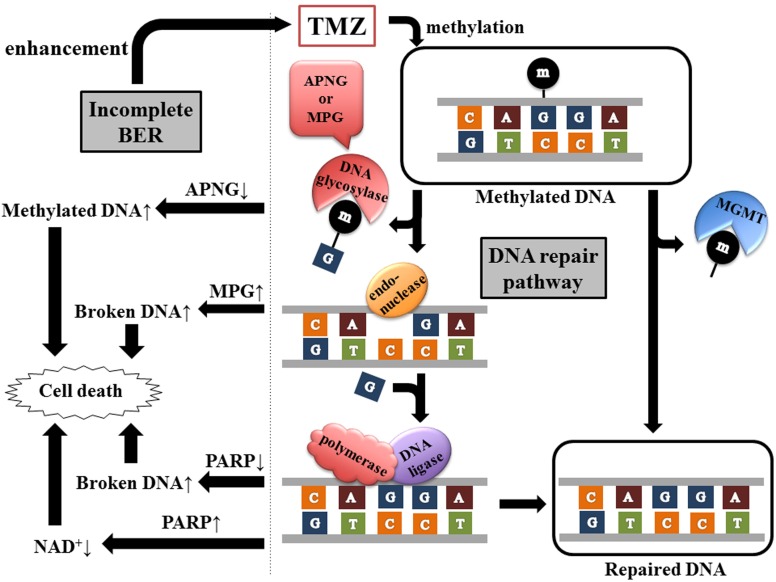
**Two pathways of methylated DNA repair.** TMZ generates a spectrum of DNA lesions including O^6^-methylguanine, N^3^-methyladenine, and N^7^-methylguanine. MGMT eliminates directly the methyl group from O^6^-methylguanine, whereas BER pathway includes multistep reaction by DNA glycosylase (APNG or MPG), endonuclease, polymerase, and DNA ligase. DNA glycosylase recognizes and removes the damaged bases. The abasic site is then hydrolyzed by endonuclease, resulting in the incision of the damaged DNA strand. Polymerase inserts a single nucleotide and DNA ligase completes the repair process. PARP, one of the polymerases of BER, catalyzes the transfer of ADP-ribose units from NAD^+^ to target proteins including PARP itself. Therefore, inhibition or hyperactivation of PARP leads to accumulation of broken DNA or NAD^+^ depletion respectively, consequently inducing cell death.

Several aspects of TMZ sensitivity cannot be explained by MGMT promoter methylation status alone. Actually, the majority of TMZ-induced methylation sites are the N^7^ position of guanine (>70%) and N^3^ position of adenine (9.2%), whereas O^6^ methylation of guanine directly removed by MGMT is the least frequent adduct (5%). N^7^-methylguanine and N^3^-methyladenine are substrates for the base excision repair (BER) system, which consists of multicatalysis reactions by a wide variety of DNA glycosylases, endonucleases, polymerases, and DNA ligases (Figure 2).

### APNG and GATA4

The BER enzyme, alkylpurine-DNA-N-glycosylase (APNG), which repairs the cytotoxic lesions N^3-methyladenine and N^7-methylguanine^, contributes to TMZ resistance. Silencing of APNG in TMZ-resistant GBM cell lines that express MGMT and APNG attenuated the repair of TMZ-induced DNA damage and enhanced apoptosis. Accordingly, exogenous expression of APNG in TMZ-sensitive GBM lines conferred resistance to TMZ *in vitro* and *in vivo* (Agnihotri et al., 2012), confirming that APNG contributes to TMZ resistance in GBM.

The GATA transcription factor family consists of six members, which bind to a consensus DNA-binding element. Recently, it was reported that GATA4 is expressed in the embryonic and adult central nervous system, inhibits astrocyte proliferation, and acts as a tumor suppressor in GBM (Agnihotri et al., 2009). Loss of GATA4, observed in the majority of GBM, was a negative prognostic marker. Re-expression of GATA4 conferred sensitivity of GBM cells to TMZ, which was independent of MGMT (Agnihotri et al., 2011). However, GATA4 reduced the expression of APNG, suggesting that GATA4 increases the anticancer potency of TMZ in human GBM cells.

### MPG, Polβ, PARP, NAD^+^ biosynthesis

Overexpression of the BER-initiating enzyme N-methylpurine DNA glycosylase (MPG) greatly enhances the effect of TMZ through the imbalance of DNA repair, independent of the MGMT status (Fishel et al., 2003; Goellner et al., 2011). Increased MPG activity induced single-strand and double-strand breaks in DNA, leading to apoptotic cell death (Dobson et al., 2000). However, these effects were abrogated by elevated expression of the rate-limiting BER enzyme DNA polymerase β (Polβ) (Tang et al., 2011). Thus, the expression levels of MPG and Polβ in tumors may represent biomarkers for alkylator therapy.

Poly (ADP-ribose) polymerase (PARP) is involved in DNA repair. PARP1 particularly has the dual effect. The cytotoxic effect of alkylating agents is enhanced by either its inhibition or hyperactivation. Inhibition of PARP1 accumulates broken DNA in the cells, resulting in cell death (Figure 2). PARP1 is the primary enzyme catalyzing the transfer of ADP-ribose units from nicotinamide adenine dinucleotide (NAD+) to target proteins including itself. Therefore, PARP1 hyperactivation leads to depletion of NAD^+^ and ATP, which are necessary for cell metabolism, followed by cell death (Tang et al., 2010) (Figure 2).

Inhibition of NAD^+^ biosynthesis also potentiates alkylator-induced cytotoxicity (Figure 2). Therefore, dual inhibition of BER and NAD^+^ biosynthesis might sensitize glioma cells to TMZ, independent of MGMT status (Goellner et al., 2011).

### Resveratrol

Resveratrol (3,5,4′-trihydroxy-trans-stilbene) is a stilbenoid found in the rinds of red grapes and in other fruits. It exhibits antioxidant, anticancer, cardioprotective, and anti-aging effects, among others. Importantly, resveratrol has been identified as a potential cancer chemopreventive agent based on its ability to modulate cancer initiation, promotion, and progression. Resveratrol increases TMZ efficacy and although the influence of resveratrol on MGMT is unknown, the combination of TMZ and resveratrol has a synergistic effect *in vitro* and *in vivo* (Lin et al., 2012). TMZ induces both apoptotic cell death and cytoprotective autophagy through a reactive oxygen species (ROS) burst and extracellular signal-regulated kinase (ERK) activation. Resveratrol possesses antioxidant or pro-oxidant activity (Holme and Pervaiz, 2007). It is possible that resveratrol suppresses TMZ-induced ROS/ERK-mediated autophagy, resulting in increased apoptosis. These data indicate that resveratrol may improve the efficacy of TMZ in patients with GBM.

## Unknown mechanisms

### Valproic ACID

Valproic acid (VPA) is a commonly prescribed AED for the treatment and prevention of seizures in patients with glioma. In addition to its antiseizure properties, VPA inhibits cell proliferation and induces differentiation and apoptosis in cancer cells (Gottlicher et al., 2001; Li et al., 2005).

Patients with GBM who were treated with VPA appeared to have a better outcome than did those not treated with VPA or treated with other AEDs (Weller et al., 2011). At least four mechanisms underlying the anti-tumor effect of VPA may be considered. First, HDAC inhibition by VPA promotes histone acetylation that loosens up the chromatin structure, consequently increasing DNA accessibility to anticancer drugs such as TMZ and enhancing the effect of γ-radiation (Van Nifterik et al., 2012). Second, concurrent treatment with VPA and TMZ synergistically induces apoptosis *in vitro* independent of the p53 status (Chen et al., 2011). Third, VPA induces autophagy, which is a caspase-independent process characterized by the accumulation of autophagic vacuoles in the cytoplasm, accompanied by extensive degradation of organelles (Fu et al., 2010). VPA sensitizes melanoma cells to TMZ *in vitro* and *in vivo* by activating the apoptotic cascade. This effect is significantly increased by INF-β (Roos et al., 2011). Fourth, VPA increases the bioavailability of TMZ by reducing the clearance of the metabolite that methylates DNA (www.temodar.com).

Therefore, VPA may affect the survival of glioma patients via different pathways.

### Ribonucleotide reductase inhibitors

Biosynthesis of deoxyribonucleotides (dNTP) from ribonucleotides is an essential step in DNA synthesis and cell replication. Ribonucleotide reductase (RR), the rate-limiting enzyme of this pathway, appears to be a negative predictive factor in patients with cancer (Jordheim et al., 2011). Several drugs that inhibit RR, such as hydroxyurea, gemcitabine, and fludarabine are used for the treatment of a variety of cancers. Among the RR inhibitors, didox (DX), and trimidox (TX) are the most potent enzyme inhibitors that demonstrated excellent anticancer activity in animal tumor models (Fritzer-Szekeres et al., 2000) and enhanced alkylator-induced cytotoxicity *in vitro* (Horvath et al., 2004). Depletion or imbalance of dNTPs by RR inhibitors may potentiate TMZ-induced cytotoxicity independent of the p53 status (Figul et al., 2003). Since most gliomas have a defective p53-mediated pathway, the ability of RR inhibitors to induce apoptosis in a p53-independent manner is an asset (Dehais et al., 2006; Masica and Karchin, 2011). In addition, RR inhibitors may enhance the radiosensitivity of the tumor by inducing G1/S arrest and/or by inhibiting DNA repair (Barker et al., 2006; Zuckerman et al., 2011). Inhibition of DNA synthesis by RR inhibitors may increase the time available for DNA repair in response to TMZ-mediated damage.

In addition to synergistic cytotoxicity, DX and TX can decrease the optimal dose of TMZ (Figul et al., 2003), thereby reducing potential adverse effects, such as myelosuppression.

## Prospective

Recent investigations have increased the understanding of the pharmacological mechanisms of TMZ. It is likely that drugs and mechanisms other than those discussed above modulate TMZ efficacy. One such mechanism is the inhibition of GSK3β, which is a serine/threonine kinase that is overexpressed and activated in GBM. We have previously reported that GSK3β inhibitor sensitized GBM cells to TMZ-induced apoptosis *in vitro* via an unknown mechanism (Miyashita et al., 2009). Notably, VPA has been shown to inhibit the activity of GSK3β (Chen et al., 1999), suggesting that GSK3β inhibition is one of the mechanisms by which VPA enhances the therapeutic efficiency of TMZ.

At present, TMZ plays a central role in the treatment of GBM. Future studies on the drugs that enhance TMZ activity without side effects are warranted, as adjuncts to standard chemotherapies can improve therapeutic benefit and prolong survival.

## Funding

This work was supported by Grant-in-Aid for Scientific Research (C-23592117) from the Ministry of Education, Culture, Sports, Science, and Technology and from the Japan Society for the Promotion of Science (Mitsutoshi Nakada) and Osaka Cancer Research Foundation (Mitsutoshi Nakada).

### Conflict of interest statement

The authors declare that the research was conducted in the absence of any commercial or financial relationships that could be construed as a potential conflict of interest.

## References

[B1] Agnihotri S. Gajadhar A. S. Ternamian C. Gorlia T. Diefes K. L. Mischel P. S. Kelly J. McGown G. Thorncroft M. Carlson B. L. Sarkaria J. N. Margison G. P. Aldape K. Hawkins C. Hegi M. Guha A. (2012). Alkylpurine-DNA-N-glycosylase confers resistance to temozolomide in xenograft models of glioblastoma multiforme and is associated with poor survival in patients. J. Clin. Invest. 122, 253–266 10.1172/JCI59334 22156195 PMC3248301

[B2] Agnihotri S. Wolf A. Munoz D. M. Smith C. J. Gajadhar A. Restrepo A. Clarke I. D. Fuller G. N. Kesari S. Dirks P. B. McGlade C. J. Stanford W. L. Aldape K. Mischel P. S. Hawkins C. Guha A. (2011). A GATA4-regulated tumor suppressor network represses formation of malignant human astrocytomas. J. Exp. Med. 208, 689–702 10.1084/jem.20102099 21464220 PMC3135351

[B3] Agnihotri S. Wolf A. Picard D. Hawkins C. Guha A. (2009). GATA4 is a regulator of astrocyte cell proliferation and apoptosis in the human and murine central nervous system. Oncogene 28, 3033–3046 10.1038/onc.2009.159 19543315

[B4] Barker C. A. Burgan W. E. Carter D. J. Cerna D. Gius D. Hollingshead M. G. Camphausen K. Tofilon P. J. (2006). *in vitro* and *in vivo* radiosensitization induced by the ribonucleotide reductase inhi-bitor Triapine (3-aminopyridine-2-carboxaldehyde-thiosemicarbazone). Clin. Cancer Res. 12, 2912–2918 10.1158/1078-0432.CCR-05-2860 16675588

[B5] Bobola M. S. Tseng S. H. Blank A. Berger M. S. Silber J. R. (1996). Role of O6-methylguanine-DNA methyltransferase in resistance of human brain tumor cell lines to the clinically relevant methylating agents temozolomide and streptozotocin. Clin. Cancer Res. 2, 735–741 9816224

[B6] Bobustuc G. C. Baker C. H. Limaye A. Jenkins W. D. Pearl G. Avgeropoulos N. G. Konduri S. D. (2010). Levetiracetam enhances p53-mediated MGMT inhibition and sensitizes glioblastoma cells to temozolomide. Neuro Oncol. 12, 917–927 10.1093/neuonc/noq044 20525765 PMC2940696

[B7] Borden E. C. Sen G. C. Uze G. Silverman R. H. Ransohoff R. M. Foster G. R. Stark G. R. (2007). Interferons at age 50, past, current and future impact on biomedicine. Nat. Rev. Drug Discov. 6, 975–990 10.1038/nrd2422 18049472 PMC7097588

[B8] Cardile V. Pavone A. Gulino R. Renis M. Scifo C. Perciavalle V. (2003). Expression of brain-derived neurotrophic factor (BDNF) and inducible nitric oxide synthase (iNOS) in rat astrocyte cultures treated with Levetiracetam. Brain Res. 976, 227–233 10.1016/S0006-8993(03)02720-3 12763257

[B9] Chen C. H. Chang Y. J. Ku M. S. Chung K. T. Yang J. T. (2011). Enhancement of temozolomide-induced apoptosis by valproic acid in human glioma cell lines through redox regulation. J. Mol. Med. 89, 303–315 10.1007/s00109-010-0707-1 21340685

[B10] Chen G. Huang L. D. Jiang Y. M. Manji H. K. (1999). The mood-stabilizing agent valproate inhibits the activity of glycogen synthase kinase-3. J. Neurochem. 72, 1327–1330 10.1046/j.1471-4159.2000.0721327.x 10037507

[B11] Dehais C. Laigle-Donadey F. Marie Y. Kujas M. Lejeune J. Benouaich-Amiel A. Pedretti M. Polivka M. Xuan K. H. Thillet J. Delattre J. Y. Sanson M. (2006). Prognostic stratification of patients with anaplastic gliomas according to genetic profile. Cancer 107, 1891–1897 10.1002/cncr.22211 16986124

[B12] Dobson A. W. Xu Y. Kelley M. R. Ledoux S. P. Wilson G. L. (2000). Enhanced mitochondrial DNA repair and cellular survival after oxidative stress by targeting the human 8-oxoguanine glycosylase repair enzyme to mitochondria. J. Biol. Chem. 275, 37518–37523 10.1074/jbc.M000831200 10982789

[B13] Dolan M. E. Mitchell R. B. Mummert C. Moschel R. C. Pegg A. E. (1991). Effect of O6-benzylguanine analogues on sensitivity of human tumor cells to the cytotoxic effects of alkylating agents. Cancer Res. 51, 3367–3372 1647266

[B14] Eyal S. Yagen B. Sobol E. Altschuler Y. Shmuel M. Bialer M. (2004). The activity of antiepileptic drugs as histone deacetylase inhibitors. Epilepsia 45, 737–744 10.1111/j.0013-9580.2004.00104.x 15230695

[B15] Figul M. Soling A. Dong H. J. Chou T. C. Rainov N. G. (2003). Combined effects of temozolomide and the ribonucleotide reductase inhibitors didox and trimidox in malignant brain tumor cells. Cancer Chemother. Pharmacol. 52, 41–46 10.1007/s00280-003-0611-2 12690517

[B16] Fishel M. L. Seo Y. R. Smith M. L. Kelley M. R. (2003). Imbalancing the DNA base excision repair pathway in the mitochondria; targeting and overexpressing N-methylpurine DNA glycosylase in mitochondria leads to enhanced cell killing. Cancer Res. 63, 608–615 12566303

[B17] Friedman H. S. Dolan M. E. Pegg A. E. Marcelli S. Keir S. Catino J. J. Bigner D. D. Schold S. C. Jr. (1995). Activity of temozolomide in the treatment of central nervous system tumor xenografts. Cancer Res. 55, 2853–2857 7796412

[B18] Fritzer-Szekeres M. Grusch M. Luxbacher C. Horvath S. Krupitza G. Elford H. L. Szekeres T. (2000). Trimidox, an inhibitor of ribonucleotide reductase, induces apoptosis and activates caspases in HL-60 promyelocytic leukemia cells. Exp. Hematol. 28, 924–930 10989193 10.1016/s0301-472x(00)00484-7

[B19] Fu J. Shao C. J. Chen F. R. Ng H. K. Chen Z. P. (2010). Autophagy induced by valproic acid is associated with oxidative stress in glioma cell lines. Neuro Oncol. 12, 328–340 10.1093/neuonc/nop005 20308311 PMC2940599

[B20] Goellner E. M. Grimme B. Brown A. R. Lin Y. C. Wang X. H. Sugrue K. F. Mitchell L. Trivedi R. N. Tang J. B. Sobol R. W. (2011). Overcoming temozolomide resistance in glioblastoma via dual inhibition of NAD+ biosynthesis and base excision repair. Cancer Res. 71, 2308–2317 10.1158/0008-5472.CAN-10-3213 21406402 PMC3077901

[B21] Gottlicher M. Minucci S. Zhu P. Kramer O. H. Schimpf A. Giavara S. Sleeman J. P. Lo Coco F. Nervi C. Pelicci P. G. Heinzel T. (2001). Valproic acid defines a novel class of HDAC inhibitors inducing differentiation of transformed cells. EMBO J. 20, 6969–6978 10.1093/emboj/20.24.6969 11742974 PMC125788

[B22] Hegi M. E. Diserens A. C. Gorlia T. Hamou M. F. De Tribolet N. Weller M. Kros J. M. Hainfellner J. A. Mason W. Mariani L. Bromberg J. E. Hau P. Mirimanoff R. O. Cairncross J. G. Janzer R. C. Stupp R. (2005). MGMT gene silencing and benefit from temozolomide in glioblastoma. N. Engl. J. Med. 352, 997–1003 10.1056/NEJMoa043331 15758010

[B23] Holme A. L. Pervaiz S. (2007). Resveratrol in cell fate decisions. J. Bioenerg. Biomembr. 39, 59–63 10.1007/s10863-006-9053-y 17308975

[B24] Horvath Z. Hochtl T. Bauer W. Fritzer-Szekeres M. Elford H. L. Szekeres T. Tihan T. (2004). Synergistic cytotoxicity of the ribonucleotide reductase inhibitor didox (3, 4-dihydroxy-benzohydroxamic acid) and the alkylating agent carmustine (BCNU) in 9L rat gliosarcoma cells and DAOY human medulloblastoma cells. Cancer Chemother. Pharmacol. 54, 139–145 10.1007/s00280-004-0795-0 15133626

[B25] Jordheim L. P. Seve P. Tredan O. Dumontet C. (2011). The ribonucleotide reductase large subunit (RRM1) as a predictive factor in patients with cancer. Lancet Oncol. 12, 693–702 10.1016/S1470-2045(10)70244-8 21163702

[B26] Li X. N. Shu Q. Su J. M. Perlaky L. Blaney S. M. Lau C. C. (2005). Valproic acid induces growth arrest, apoptosis, and senescence in medulloblastomas by increasing histone hyperacetylation and regulating expression of p21Cip1, CDK4, and CMYC. Mol. Cancer Ther. 4, 1912–1922 10.1158/1535-7163.MCT-05-0184 16373706

[B27] Lin C. J. Lee C. C. Shih Y. L. Lin T. Y. Wang S. H. Lin Y. F. Shih C. M. (2012). Resveratrol enhances the therapeutic effect of temozolomide against malignant glioma *in vitro* and *in vivo* by inhibiting autophagy. Free Radic. Biol. Med. 52, 377–391 10.1016/j.freeradbiomed.2011.10.487 22094224

[B28] Masica D. L. Karchin R. (2011). Correlation of somatic mutation and expression identifies genes important in human glioblastoma progression and survival. Cancer Res. 71, 4550–4561 10.1158/0008-5472.CAN-11-0180 21555372 PMC3129415

[B29] Miyashita K. Kawakami K. Nakada M. Mai W. Shakoori A. Fujisawa H. Hayashi Y. Hamada J. Minamoto T. (2009). Potential therapeutic effect of glycogen synthase kinase 3beta inhibition against human glioblastoma. Clin. Cancer Res. 15, 887–897 10.1158/1078-0432.CCR-08-0760 19188159

[B30] Motomura K. Natsume A. Kishida Y. Higashi H. Kondo Y. Nakasu Y. Abe T. Namba H. Wakai K. Wakabayashi T. (2011). Benefits of interferon-beta and temozolomide combination therapy for newly diagnosed primary glioblastoma with the unmethylated MGMT promoter: a multicenter study. Cancer 117, 1721–1730 10.1002/cncr.25637 21472719

[B31] Natsume A. Ishii D. Wakabayashi T. Tsuno T. Hatano H. Mizuno M. Yoshida J. (2005). IFN-beta down-regulates the expression of DNA repair gene MGMT and sensitizes resistant glioma cells to temozolomide. Cancer Res. 65, 7573–7579 10.1158/0008-5472.CAN-05-0036 16140920

[B32] Quinn J. A. Jiang S. X. Reardon D. A. Desjardins A. Vredenburgh J. J. Rich J. N. Gururangan S. Friedman A. H. Bigner D. D. Sampson J. H. McLendon R. E. Herndon J. E. 2nd Walker A. Friedman H. S. (2009). Phase II trial of temozolomide plus o6-benzylguanine in adults with recurrent, temozolomide-resistant malignant glioma. J. Clin. Oncol. 27, 1262–1267 10.1200/JCO.2008.18.8417 19204199 PMC2667825

[B33] Roos W. P. Jost E. Belohlavek C. Nagel G. Fritz G. Kaina B. (2011). Intrinsic anticancer drug resistance of malignant melanoma cells is abrogated by IFN-beta and valproic acid. Cancer Res. 71, 4150–4160 10.1158/0008-5472.CAN-10-3498 21493591

[B34] Strahl B. D. Allis C. D. (2000). The language of covalent histone modifications. Nature 403, 41–45 10.1038/47412 10638745

[B35] Tang J. B. Goellner E. M. Wang X. H. Trivedi R. N. St Croix C. M. Jelezcova E. Svilar D. Brown A. R. Sobol R. W. (2010). Bioenergetic metabolites regulate base excision repair-dependent cell death in response to DNA damage. Mol. Cancer Res. 8, 67–79 10.1158/1541-7786.MCR-09-0411 20068071 PMC2808464

[B36] Tang J. B. Svilar D. Trivedi R. N. Wang X. H. Goellner E. M. Moore B. Hamilton R. L. Banze L. A. Brown A. R. Sobol R. W. (2011). N-methylpurine DNA glycosylase and DNA polymerase beta modulate BER inhibitor potentiation of glioma cells to temozolomide. Neuro Oncol. 13, 471–486 10.1093/neuonc/nor011 21377995 PMC3093332

[B37] Ueda Y. Doi T. Takaki M. Nagatomo K. Nakajima A. Willmore L. J. (2009). Levetiracetam enhances endogenous antioxidant in the hippocampus of rats: *in vivo* evaluation by brain microdialysis combined with ESR spectroscopy. Brain Res. 1266, 1–7 10.1016/j.brainres.2009.02.040 19268434

[B38] Van Nifterik K. A. Van Den Berg J. Slotman B. J. Lafleur M. V. Sminia P. Stalpers L. J. (2012). Valproic acid sensitizes human glioma cells for temozolomide and gamma-radiation. J. Neurooncol. 107, 61–67 10.1007/s11060-011-0725-z 22037799

[B39] Wakabayashi T. Kayama T. Nishikawa R. Takahashi H. Hashimoto N. Takahashi J. Aoki T. Sugiyama K. Ogura M. Natsume A. Yoshida J. (2011). A multicenter phase I trial of combination therapy with interferon-beta and temozolomide for high-grade gliomas (INTEGRA study): the final report. J. Neurooncol. 104, 573–577 10.1007/s11060-011-0529-1 21327711

[B40] Weller M. Gorlia T. Cairncross J. G. Van Den Bent M. J. Mason W. Belanger K. Brandes A. A. Bogdahn U. Macdonald D. R. Forsyth P. Rossetti A. O. Lacombe D. Mirimanoff R. O. Vecht C. J. Stupp R. (2011). Prolonged survival with valproic acid use in the EORTC/NCIC temozolomide trial for glioblastoma. Neurology 77, 1156–1164 10.1212/WNL.0b013e31822f02e1 21880994 PMC3265044

[B41] Yoshino A. Ogino A. Yachi K. Ohta T. Fukushima T. Watanabe T. Katayama Y. Okamoto Y. Naruse N. Sano E. (2009). Effect of IFN-beta on human glioma cell lines with temozolomide resistance. Int. J. Oncol. 35, 139–148 10.3892/ijo_00000322 19513561

[B42] Zuckerman J. E. Hsueh T. Koya R. C. Davis M. E. Ribas A. (2011). siRNA knockdown of ribonucleotide reductase inhibits melanoma cell line proliferation alone or synergistically with temozolomide. J. Invest. Dermatol. 131, 453–460 10.1038/jid.2010.310 20944646

